# Breast Disease Patterns Among Patients Presenting for Mammography in a Major Hospital in the Volta Region of Ghana: A Five-Year Descriptive Retrospective Study

**DOI:** 10.1155/ijbc/5542692

**Published:** 2025-06-30

**Authors:** Seth Kwadjo Angmorterh, Manuel Betancourt Benjamin, Rhoda Kokwe Oppong, Patience Nyamekye Agyemang, Nathaniel Awentiirin Angaag, Kafui Kossi Kekessie, Riaan van de Venter, John Nsor-Atindana, Cosmos Yarfi, Portia Mamle Angmorterh, Sonia Aboagye, Mariella Mawunyo Amoussou-Gohoungo, Adam Inusah, Klenam Dzefi-Tettey, Nii Korley Kortei

**Affiliations:** ^1^Department of Medical Imaging, School of Allied Health Sciences, University of Health and Allied Sciences (UHAS), Ho, Ghana; ^2^Department of Radiology, School of Medicine, University of Health and Allied Sciences (UHAS), Ho, Ghana; ^3^Department of Radiography, School of Clinical Care and Medicinal Sciences, Faculty of Health Sciences, Nelson Mandela University, Gqeberha, South Africa; ^4^Department of Nutrition and Dietetics, School of Allied Health Sciences, University of Health and Allied Sciences (UHAS), Ho, Ghana; ^5^Department of Physiotherapy and Rehabilitation Sciences, School of Allied Health Sciences, University of Health and Allied Sciences (UHAS), Ho, Ghana; ^6^Department of French Education, Faculty of Foreign Languages Education, University of Education, Winneba, Ghana; ^7^Department of Speech, Language and Hearing Sciences, School of Allied Health Sciences, University of Health and Allied Sciences (UHAS), Ho, Ghana; ^8^Department of Radiology, Korle-Bu Teaching Hospital, Accra, Ghana; ^9^Department of Sports Nutrition, School of Sports and Exercise Medicine, University of Health and Allied Sciences, Ho, Ghana

**Keywords:** breast cancer, diagnostic mammography, Ghana, screening mammography, Volta region

## Abstract

**Introduction:** The practice of mammography has transitioned from analog to digital with improved accuracy and significant changes to findings. This study was aimed at investigating the current patterns of breast diseases among women presenting for mammography at a major hospital in the Volta region of Ghana.

**Methods:** This descriptive retrospective study reviewed 508 mammography and complimentary breast ultrasound reports conducted between October 2019 and May 2023. Because they were incomplete and had essential patient data missing, 28 reports (*n* = 28) were excluded. Data extracted from the reports included patients' age, clinical indication, breast density, imaging impression, and BI-RADS classification for each breast. Data were analyzed using the Statistical Package for the Social Sciences (SPSS) Version 26, and results are presented using descriptive and inferential statistics.

**Results:** The study involved 480 women, aged 40–86 years (mean = 54.6 ± 10.1). The distribution of the breast densities of the women was as follows: almost entirely fatty (*n* = 79, 16.46%), scattered areas of fibroglandular density (*n* = 226, 47.08%), heterogeneously dense (*n* = 145, 30.21%), and extremely dense (*n* = 30, 6.25%). There was a statistically significant association between age and breast density (*p* < 0.01). While 30 (6.25%) of the women presented for screening, 450 (93.75%) presented for diagnostic mammography. Breast pain (*n* = 189, 39.38%), breast lump/mass (*n* = 155, 32.29%), and suspected breast cancer (*n* = 47, 9.79%) were the most common clinical indications. The study recorded a total of 960 BI-RADS classifications of which 261 (27.19%) were negative and 699 (72.81%) were positive. Most of the positive findings (*n* = 521, 74.54%) were BI-RADS 2 and 3. Both benign and suspicious for malignancy or highly suggestive of malignancy lesions were common across women aged 40–50 years. There was a statistically significant association between age and BI-RADS classification (*p* < 0.01).

**Conclusion:** This study showed that most of the women presented for diagnostic mammography. Attendance for screening mammography was poor among women presenting for mammography at the hospital; hence, women should be encouraged through health education and other campaign strategies to undergo screening mammography more regularly to facilitate more timely detection and diagnosis of breast diseases. A third of the women in our study had dense breasts. The vast majority of the women had positive findings, but the majority of these findings were indicative of benign breast diseases.

## 1. Introduction

Breast disease may present as benign conditions (e.g., fibroadenoma, lipoma, hamartoma, breast cyst, intraductal papilloma, and mastitis) or malignant conditions such as breast cancer [1–4]. Benign breast lesions are noncancerous, characterized by well-defined borders, and may commonly present as pain whereas malignant breast lesions are cancerous, characterized by irregular and poor marginal definitions [1, 5, 6]. Approximately 2.3 million breast cancer cases have been recorded globally, with 190,000 of these in Africa [6]. In Ghana, breast cancer accounts for over 30% of cancer incidence and forms the majority of cancer-related deaths [7]. The breast cancer incidence-to-mortality ratio indicates higher statistics for low- and middle-income countries such as Ghana due to the absence of national breast screening programs, inadequate knowledge about the disease, poverty and poor health infrastructure, and systems for early detection and effective treatment [8, 9]. Similarly, sociocultural beliefs and influences are important barriers to timeous diagnosis and treatment of breast cancer in Africa [10, 11]. Breast evaluation using mammography aids in the early detection of breast diseases [12]. Mammography is categorized into two—screening and diagnostic. Screening mammography involves asymptomatic patients, whereas diagnostic mammography involves patients presenting with signs and symptoms indicative of breast disease such as pain, breast mass, discharge, burning sensations, swellings, and ulcerations [13, 14].

To ensure global standardization, mammography is reported based on the American College of Radiology (ACR) Breast Imaging Reporting and Data System (BI-RADS). The BI-RADS is a risk assessment and quality assurance (QA) tool that provides a widely accepted lexicon and reporting schema for breast imaging. The BI-RADS provides classifications for breast density and findings. The BI-RADS breast density classifications are four, including, almost entirely fatty, scattered areas of fibroglandular density, heterogeneously dense, and extremely dense. The BI-RADS breast findings' categorization ranges from 0 (*incomplete*), 1 (*negative*), 2 (*benign*), 3 (*probably benign*), 4a (*low suspicion for malignancy*), 4b (*intermediate suspicion for malignancy*), 4c (*moderate suspicion for malignancy*), 5 (*highly suggestive of malignancy*), and 6 (*known biopsy proven malignancy*) [2].

Several studies have investigated screening and diagnostic mammography findings and their associated cancer risks across different countries and clinical settings [1, 14–17]. For instance, a study by Alquimim et al. [16] among patients presenting for screening and diagnostic mammography in Brazil shows that the majority of them had benign pathologies. A review of screening and diagnostic mammography examinations in Nigeria showed diverse clinical findings: cysts, fibroadenomas, lymphadenopathy, and malignancy, with almost 5% of the lesions being malignant and 30% being benign [15]. In Ghana, Brakohiapa et al. [1] indicated that pain was the most common clinical indication across patients presenting for screening and diagnostic mammography, and the majority of the breast lesions were benign. Another Ghanaian study by Edzie et al. [14] recorded diverse mammography findings including axillary lymph nodes, macrocalcification, and masses. In a nationwide Ghanaian study, Ghartey et al. [17] indicated that the Volta region, mainly a rural part of the country (located in Southern Ghana), recorded the highest prevalence of breast cancer.

Although the findings of Ghartey et al. [17] provide useful information on breast diseases across the Volta region of Ghana, they may not reflect the current trend because this study was conducted between 2007 and 2008, more than 15 years ago. Similarly, mammography practice has transitioned from analog to digital over the years with improved accuracy and significant changes to findings. To the authors' knowledge, there is no current study on this subject in the Volta region. This study is therefore aimed at investigating the current patterns of breast diseases among women presenting for mammography at the Ho Teaching Hospital (HTH) in the Volta region of Ghana. Although this is a single-center study and the findings may not be generalizable to the entire country, healthcare professionals and stakeholders may cautiously implement the findings of this study in policy formulation and strategic planning to improve breast cancer awareness, screening campaigns and other associated services in Ghana. Also, the findings of this study will provide baseline data to inform a nationwide study on the patterns of breast diseases in Ghana.

## 2. Methods

### 2.1. Study Design and Setting

This study involved a retrospective descriptive review of mammography and complementary breast ultrasonography performed at the Radiology Department of the HTH in the Volta region of Ghana between October 2019 and May 2023. The 300-bed capacity hospital provides a wide range of services, including Outpatient, Inpatient, Mental Health, Dialysis, Orthopedic, and Radiology. This hospital was selected because it is the only hospital equipped with mammography equipment in the Volta region of Ghana at the time.

### 2.2. Inclusion and Exclusion Criteria

The reports of all patients who underwent mammography and complementary breast ultrasonography between the period under review were included in the study. However, reports with no patient age, clinical indication, breast density classification, clinical impression, and BI-RADS classification were excluded.

### 2.3. Data Retrieval Process

The mammography and complimentary breast ultrasonography reports were retrieved from the radiology archives of the hospital. The mammography examinations were performed by a radiographer with over 5 years work experience, and the complementary ultrasonography together with the mammography reporting was performed by a radiologist with over 8 years work experience. The mammography reports were written in accordance with the ACR BI-RADS. The breast densities of the women were assessed subjectively by the radiologist and classified into four—almost entirely fatty, scattered fibroglandular, heterogeneously dense, and extremely dense. Then, 508 mammography and complimentary ultrasonography reports (*n* = 508) were retrieved for the period under review. However, following data examination and cleaning, 28 reports were excluded because they met the exclusion criteria. Data extracted from the reports included patients' age, clinical indication, breast density, imaging impression, and BI-RADS classification for both breasts.

### 2.4. Ethical Considerations

The Research Ethics Committee (REC) of the University of Health and Allied Sciences granted approval for the study (UHAS-REC A.8 [119] 22–23). Specific consent was waived due to the retrospective nature of the study. To ensure confidentiality, the reports were de-identified and made available only to the researchers. The study conformed with the Declaration of Helsinki and Belmont Report.

### 2.5. Statistical Analysis

**Figure 1:**
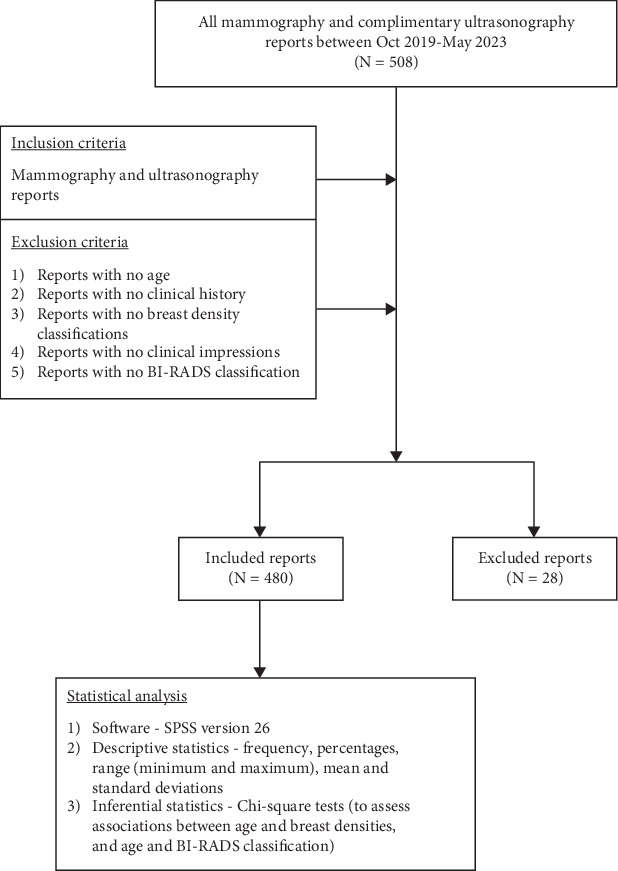
Flow diagram of study methodology.

Data obtained were inputted into the Statistical Package for the Social Sciences (SPSS) Version 26. Descriptive statistics comprising frequencies, percentages, range (minimum and maximum), mean, and standard deviations were adopted for reporting the findings. Also, the nonparametric chi-square tests were performed to determine associations between patients' age and breast density and patents' age and BI-RADS classification for both breasts. A *p* value ≤ 0.05 was considered statistically significant. The methodology for this study is summarized in Figure 1.

## 3. Results

The study involved 480 women (*n* = 480), aged between 40 and 86 years (mean = 54.6 ± 10.1) with a modal age of 48 years. The women who underwent the mammography had different breast densities. Most of them (*n* = 226, 47.08%) had scattered fibroglandular breast densities, whereas 145 (30.21%) had heterogeneously dense breasts. As shown in Table 1, the results of the chi-square test indicated that there was a statistically significant association between age and breast density (*χ*^2^ = 49.94, *p* < 0.001).

The women who underwent the mammography examinations presented with diverse clinical indications. Breast pain (*n* = 189, 39.38%), breast lump/mass (*n* = 155, 32.29%), and suspected breast cancer (*n* = 47, 9.79%) were the top three clinical indications. Screening mammography was done for 30 (6.25%) women. The detailed clinical indications for both breasts across the patients are presented in Table 2.

**Figure 2:**
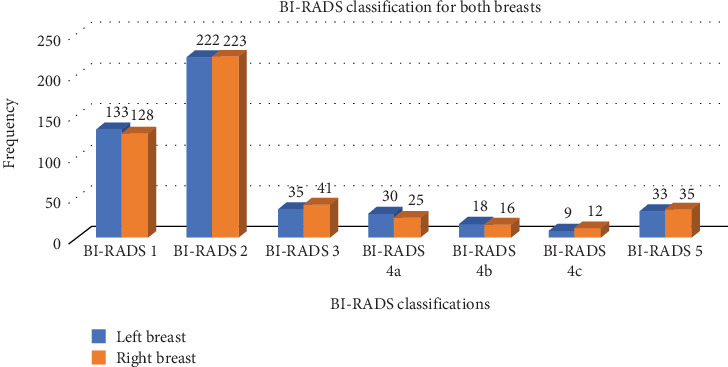
BI-RADS classification for both breasts.

BI-RADS classification was assigned to each breast across the 480 women (*n* = 480), resulting in a total of 960 BI-RADS classifications (*n* = 960). Most of the BI-RADS findings were BI-RADS 2 (left breast = 222 and right breast = 223). The BI-RADS classification of women who underwent the mammography examinations across both breasts is presented in Figure 2.

Most of the women recorded more than one clinical finding resulting in a total of 1049 findings across the 480 women. The main clinical findings that were present among the cohort of mammography reports analyzed were as follows: axillary lymph nodes (*n* = 592, 56.43%), breast mass (*n* = 162, 15.44%), intramammary lymph nodes (*n* = 82, 7.82%), and microcalcifications (*n* = 47, 4.48%). The various breast mammography findings with their corresponding BI-RADS classifications across the women who presented for mammography within the study period are shown in Table 3.

From the 960 BI-RADS classifications, the study recorded 261 (27.19%) negative and 699 (72.81%) positive findings. Out of the positive findings, the majority (*n* = 445, 63.66%) were benign (BI-RADS 2). As shown in Table 4, the results of chi-square test indicated that there was a statistically significant association between age and BI-RADS classification (*χ*^2^ = 32.73, *p* < 0.001).

## 4. Discussion

The aim of this study was to retrospectively review mammography and complimentary breast ultrasonography reports between October 2019 and May 2023 to describe the current patterns of breast diseases among women presenting for mammography at a major hospital in the Volta region of Ghana. This study included 480 women with a mean age of 54.6 ± 10.1 years, consistent with other Ghanaian mammography-related studies [1, 14, 18, 19]. The findings of this study showed that only 30 (6.25%) of the women presented for screening mammography, whereas 450 (93.75%) presented for diagnostic mammography. These findings are inconsistent with previous Ghanaian studies [1, 14] which recorded more patients presenting for screening than diagnostic mammography. The reason for the inconsistency can be attributed to the study locations. The studies by Brakohiapa et al. [1] and Edzie et al. [14] were conducted in Accra (the capital city) and Cape Coast (a major tourism hub), respectively. Compared to the Volta region, the setting for this current study, Accra and Cape Coast receive greater support and patronage of breast cancer awareness programs due to the presence of media and health-related nongovernmental organizations (NGOs) [20].

The low number of patients presenting for screening mammography recorded in our study could also be due to the lack of a national breast cancer screening program in Ghana and the absence of comprehensive financing arrangements for patients presenting for mammography. For instance, mammography examinations in Ghana are not covered under the National Health Insurance Scheme (NHIS)—the public-funded health insurance. The Volta region is considered rural, inhabited by several women of low economic status, and therefore, these women may find mammography unaffordable [21]. Notwithstanding that, sociocultural beliefs and practices may also be significant barriers to timely detection and diagnosis of breast diseases because women may fear ostracization due to the negative connotations attached to breast cancer [10, 11]. Consequently, most women in the Volta region are more likely to present with late-stage breast cancer and other breast diseases, thereby reducing the effectiveness of treatment and their chances of survival [22]. To improve participation in screening mammography, it is recommended that a comprehensive national breast screening program in Ghana must be developed. Mass and small media, public education, reduction of economic and structural barriers, client reminders, and engagement of health professionals to promote awareness about breast diseases should also be considered [9, 23].

In this study, 175 (36.46%) of the women who presented for mammography had dense breasts—heterogeneously and extremely dense. The majority (*n* = 102, 58.29%) of the women with dense breasts were aged 40–50 years. The finding about dense breasts is higher than the findings of Galukande and Kiguli-Malwadde [24], conducted in Uganda, which indicated that almost 26% of women who presented for mammography examination had dense breasts. The clinical implication of 175 (36.46%) women having dense breasts in the current study is that the sensitivity of mammography will significantly reduce across these women because denser breast parenchyma may increase the chance of missing small breast lesions, thereby increasing the risk of breast cancer [18, 25]. Another clinical implication of women having dense breasts is that stand-alone mammography may not be enough to provide comprehensive diagnostic information, and that there may be the need for complementary breast ultrasound examinations as part of the clinical imaging pathway for women with dense breasts in other hospitals in Ghana.

In this study, BI-RADS classification was assigned to each breast across the 480 women, resulting in a total of 960 BI-RADS classifications. The results of this study showed that there were similar percentage distributions between the left and right breasts, which is inconsistent with the studies conducted by Edzie et al. [14] and Thomas et al. [8] who found that the majority of the abnormalities recorded across a Ghanaian population were in the right breast. The current study recorded 261 (27.19%) negative and 699 (72.81%) positive findings. From the positive findings, the majority (*n* = 521, 74.54%) were benign (BI-RADS 2 and 3), whereas 178 (25.46%) were suspicious for malignancy or highly suggestive of malignancy (BI-RADS 4a-5). Similar to previous studies [1, 14, 15], the positive findings (both benign and malignant) recorded in our study were common across women aged 40–50 years. This evidence supports the recommendation of the Ghana Health Service (GHS)—the government agency responsible for health service provision and policy in Ghana—that screening mammography should start at 40 years. There is evidence to show that breast cancer mortality is generally reduced with screening mammography, although estimates are influenced by several factors and the extents of effect vary across patients [26]. Across all BI-RADS classifications, the presence of axillary lymph nodes was the most common finding, similar to the study conducted by Edzie et al. [14] which indicated that the most common mammography finding across women who presented for mammography examination in Cape Coast was axillary lymph nodes.

## 5. Conclusion

This study showed that most of the women in the Volta region of Ghana presented for diagnostic mammography. Attendance for screening mammography was poor among women presenting for mammography at the hospital; hence, women should be encouraged through health education and other campaign strategies to undergo screening mammography more regularly to facilitate more timely detection and diagnosis of breast diseases. A third of the women in our study had dense breasts. There was a statistically significant association between age and breast density. Breast pain, breast lump/mass, and suspected breast cancer were the most common clinical indications. The main clinical findings that were present among the cohort of mammography reports analyzed were axillary lymph nodes, breast mass, intramammary lymph nodes, and microcalcifications. The vast majority of the women had positive findings, but the majority of these findings were indicative of benign breast diseases. Most of the positive findings (both benign and malignant) recorded in our study were common among women aged 40–50 years. There was a statistically significant association between age and BI-RADS classification.

## 6. Limitation

BI-RADS 6 patients (known biopsy proven malignancy) hardly return after biopsy; hence, there is no data on them. Thus, information about this could not be investigated in this study. Also, this study involved only one hospital, so the results cannot be generalized to the entirety of Ghana.

## Figures and Tables

**Table 1:** Distribution of breast densities across different age groups.

**Variable**	**Total** **n** ** (%)**	**Age groups (years)**					**χ** ^2^/**p value**
**Breast densities**		**40–50** **n** ** (%)**	**51–60** **n** ** (%)**	**61–70** **n** ** (%)**	**71–80** **n** ** (%)**	**81–90** **n** ** (%)**	
Almost entirely fatty	79 (16.46)	22 (27.85)	26 (32.91)	18 (22.78)	9 (11.39)	4 (5.06)	*χ* ^2^ = 49.94, *p* < 0.001
Scattered areas of fibroglandular density	226 (47.08)	68 (30.09)	79 (34.96)	57 (25.22)	20 (8.85)	2 (0.88)	
Heterogeneously dense	145 (30.21)	83 (57.24)	30 (20.69)	27 (18.62)	4 (2.76)	1 (0.69)	
Extremely dense	30 (6.25)	19 (63.33)	4 (13.33)	5 (16.67)	2 (6.67)	0 (0.00)	
Total	480 (100)	192 (40.00)	139 (28.96)	107 (22.29)	35 (7.29)	7 (1.46)	

**Table 2:** Distribution of clinical indications across patients.

**Clinical indications**	**Total** **n** ** (%)**	**Both breasts** **n** ** (%)**	**Right breast** **n** ** (%)**	**Left breast** **n** ** (%)**
Breast pain	189 (39.38)	134 (70.90)	25 (13.23)	30 (15.87)
Breast lump/mass	155 (32.29)	32 (20.65)	49 (31.61)	74 (47.74)
Suspected breast cancer	47 (9.79)	0 (0.00)	19 (40.43)	28 (59.57)
Breast discharge	23 (4.79)	8 (34.78)	5 (21.74)	10 (43.48)
Screening	30 (6.25)	30 (100.00)	0 (0.00)	0 (0.00)
Breast asymmetry	22 (4.58)	0 (0.00)	13 (59.09)	9 (40.91)
Mastitis	9 (1.88)	0 (0.00)	5 (55.56)	4 (44.44)
Abscess	4 (0.83)	0 (0.00)	2 (50.00)	2 (50.00)
Breast conserving therapy	1 (0.21)	0 (0.00)	1 (100.00)	0 (0.00)
Total	480 (100.00)	204 (42.50)	119 (24.79)	157 (32.71)

**Table 3:** Mammography findings with their corresponding BI-RADS classifications.

**Clinical finding**	**Total** **n** ** (%)**	**BI-RADS 1** **n** ** (%)**	**BI-RADS 2** **n** ** (%)**	**BI-RADS 3** **n** ** (%)**	**BI-RADS 4a** **n** ** (%)**	**BI-RADS 4b** **n** ** (%)**	**BI-RADS 4c** **n** ** (%)**	**BI-RADS 5** **n** ** (%)**
Axillary lymph node	592 (56.43)	9 (1.52)	416 (70.27)	36 (6.08)	39 (6.59)	23 (3.89)	16 (2.70)	53 (8.95)
Breast mass	162 (15.44)	2 (1.23)	3 (1.85)	13 (8.02)	43 (26.54)	24 (14.81)	16 (9.88)	61 (37.65)
Intramammary lymph node	82 (7.82)	0 (0.00)	38 (46.34)	17 (20.73)	10 (12.20)	4 (4.88)	3 (3.66)	10 (12.20)
Microcalcification	47 (4.48)	0 (0.00)	11 (23.40)	11 (23.40)	4 (8.51)	5 (10.64)	4 (8.51)	12 (25.53)
Soft tissue thickening	40 (3.81)	3 (7.50)	3 (7.50)	2 (5.00)	5 (12.50)	2 (5.00)	2 (5.00)	23 (57.50)
Breast cyst	31 (2.96)	0 (0.00)	9 (29.03)	10 (32.26)	10 (32.26)	1 (3.23)	1 (3.23)	0 (0.00)
Dilated ducts	29 (2.76)	0 (0.00)	9 (31.03)	10 (34.48)	5 (17.24)	4 (13.79)	1 (3.45)	0 (0.00)
Edema	18 (1.72)	0 (0.00)	0 (0.00)	1 (5.56)	0 (0.00)	5 (27.78)	2 (11.11)	10 (55.56)
Mastitis	15 (1.43)	0 (0.00)	1 (6.67)	4 (26.67)	1 (6.67)	2 (13.33)	3 (20.00)	4 (26.67)
Papilloma	14 (1.33)	0 (0.00)	2 (14.29)	0 (0.00)	9 (64.29)	1 (7.14)	2 (14.29)	0 (0.00)
Fibroadenoma	10 (0.95)	0 (0.00)	3 (30.00)	3 (30.00)	2 (20.00)	1 (10.00)	1 (10.00)	0 (0.00)
Breast abscess	4 (0.38)	0 (0.00)	1 (25.00)	0 (0.00)	2 (50.00)	1 (25.00)	0 (0.00)	0 (0.00)
Retracted nipple	3 (0.29)	0 (0.00)	0 (0.00)	1 (33.33)	0 (0.00)	0 (0.00)	0 (0.00)	2 (66.67)
Breast asymmetry	2 (0.19)	0 (0.00)	0 (0.00)	2 (100.00)	0 (0.00)	0 (0.00)	0 (0.00)	0 (0.00)
Total	1049 (100)	14 (1.33)	496 (47.28)	110 (10.49)	130 (12.39)	73 (6.96)	51 (4.86)	175 (16.68)

**Table 4:** BI-RADS classification across different age groups.

**BI-RADS classification**	**Total** ***n* (%)**	**Age (years)**					**χ** ^2^/**p value**
		**40–50** **n** ** (%)**	**51–60** **n** ** (%)**	**61–70** **n** ** (%)**	**71–80** **n** ** (%)**	**81–90** **n** ** (%)**	
BI-RADS 1	261 (27.19)	104 (39.85)	79 (30.27)	55 (21.07)	19 (7.28)	4 (1.53)	*χ* ^2^ = 32.73, *p* < 0.001
BI-RADS 2	445 (46.35)	176 (39.55)	123 (27.64)	107 (24.04)	34 (7.64)	5 (1.12)	
BI-RADS 3	76 (7.92)	31 (40.79)	23 (30.26)	15 (19.74)	6 (7.89)	1 (1.32)	
BI-RADS 4a	55 (5.73)	31 (56.36)	12 (21.82)	9 (16.36)	2 (3.64)	1 (1.82)	
BI-RADS 4b	34 (3.54)	15 (44.12)	12 (35.29)	5 (14.71)	2 (5.88)	0 (0.00)	
BI-RADS 4c	21 (2.19)	6 (28.57)	7 (33.33)	5 (23.81)	1 (4.76)	2 (9.52)	
BI-RADS 5	68 (7.08)	21 (30.88)	22 (32.35)	18 (26.47)	6 (8.82)	1 (1.47)	

## Data Availability

The data that support the findings of this study are available from the corresponding author upon reasonable request.
